# Conditioning with Fludarabine-Busulfan versus Busulfan-Cyclophosphamide Is Associated with Lower aGVHD and Higher Survival but More Extensive and Long Standing Bone Marrow Damage

**DOI:** 10.1155/2016/3071214

**Published:** 2016-10-24

**Authors:** Xin He, YongBin Ye, XiaoJun Xu, Jing Wang, YuXian Huang, GuangYang Weng, MingWan Zhang, KunYuan Guo

**Affiliations:** ^1^Department of Hematology, Zhongshan Hospital of Sun Yat-Sen University and Zhongshan City People's Hospital, Zhongshan 528403, China; ^2^Department of Hematology, Zhujiang Hospital, Southern Medical University, Guangzhou 510282, China

## Abstract

Acute graft-versus-host disease (aGVHD) is a major complication of allogeneic hematopoietic stem cell transplantation (allo-HSCT) and a major cause of nonrelapse mortality after allo-HSCT. A conditioning regimen plays a pivotal role in the development of aGVHD. To provide a platform for studying aGVHD and evaluating the impact of different conditioning regimens, we established a murine aGVHD model that simulates the clinical situation and can be conditioned with Busulfan-Cyclophosphamide (Bu-Cy) and Fludarabine-Busulfan (Flu-Bu). In our study, BALB/c mice were conditioned with Bu-Cy or Flu-Bu and transplanted with 2 × 10^7^ bone marrow cells and 2 × 10^7^ splenocytes from either allogeneic (C57BL/6) or syngeneic (BALB/c) donors. The allogeneic recipients conditioned with Bu-Cy had shorter survivals (*P* < 0.05), more severe clinical manifestations, and higher hepatic and intestinal pathology scores, associated with increased INF-*γ* expression and diminished IL-4 expression in serum, compared to allogeneic recipients conditioned with Flu-Bu. Moreover, higher donor-derived T-cell infiltration and severely impaired B-cell development were seen in the bone marrow of mice, exhibiting aGVHD and conditioned with Flu-Bu. Our study showed that the conditioning regimen with Bu-Cy resulted in more severe aGVHD while the Flu-Bu regimen was associated with more extensive and long standing bone marrow damage.

## 1. Introduction

Allogeneic hematopoietic stem cell transplantation (allo-HSCT) is an effective treatment strategy for many hematologic and nonhematologic diseases [1]. During allo-HSCT, donor-derived immunocompetent cells play an important part in producing graft-versus-leukemia/lymphoma (GVL) effect by reestablishing the immune system of the recipient. However, these donor-derived cells may also attack the recipient's healthy organs, this is, known as graft-versus-host disease (GVHD). Acute GVHD (aGVHD) is a major cause of nonrelapse mortality, accounting for over 20% of transplantation-related mortality [2].

The pathophysiology of aGVHD involves an immunoreaction between the immunocompetent T-cells in the graft and the host's histoincompatible alloantigens, including activation of immunocytes and proinflammatory cytokines. The process has been broken down into three phases: the tissue damage phase, the T-cell priming phase, and the effector phase [3]. However, the precise role of cytokines, chemokines, and immunocytes in each phase has not been well elucidated. In order to better prevent and control aGVHD, further understanding of its pathogenesis is essential. The clinical use of immunosuppressive agents (e.g., methotrexate or calcineurin inhibitors), cytotoxic drugs, or in vitro/in vivo T-depletion can significantly reduce the morbidity and mortality of aGVHD, but it also weakens the GVL effect, resulting in a high relapse rate after transplantation [4]. Many other new immunomodulators and new strategies such as agents targeting the cytokine/chemokine-receptor interaction and other novel approaches are currently under investigation in animal models, primarily mouse models [5]. Murine models of allo-HSCT have been widely applied in elucidating the pathogenesis of aGVHD and testing new strategies and interventions in preclinical studies. In contrast to the clinical situation, conditioning regimens of most allo-HSCT models are based on total body irradiation, and reports about transplantation models conditioned with chemotherapy are rare [6]. Since most conditioning regimens in clinical situations are combination chemotherapy, establishing a transplantation model that mimics clinical practice would provide a better platform for further preclinical studies of aGVHD.

Pretransplantation conditioning provides space in the hematopoietic compartment for donor stem cells, eradicates residual leukemia/lymphoma cells, and provides immunosuppression to prevent graft failure [7]. The conditioning intensity is closely related to posttransplantation relapse and the development of aGVHD. Previous experimental studies have confirmed that pretransplantation conditioning intensity influences the severity of aGVHD by affecting the release of inflammatory cytokines [8]. Moreover, different gene expression profiles of the liver following total body-irradiated or Busulfan-Cyclophosphamide (Bu-Cy) conditioned mice have been reported [9]. The conditioning regimens Bu-Cy and Fludarabine-Busulfan (Flu-Bu) are commonly used in clinical practice. However, randomized trials comparing the two conditioning regimens have produced conflicting results regarding the incidence of aGVHD and the nonrelapse mortality of Busulfan plus Fludarabine [10, 11]. To date, no related studies have compared the aGVHD of conditioning regimens between Bu-Cy and Flu-Bu in animal models. In this study, we established a murine model of allo-HSCT conditioned with Bu-Cy or Flu-Bu and compared the aGVHD frequency and severity of the two regimens.

## 2. Materials and Methods

### 2.1. Animals

Male BALB/c (H-2Kd) and female C57BL/6 (H-2Kb) mice, 7-8 weeks old, were used as MHC-incompatible allo-HSCT recipients and donors, respectively. All mice were purchased from Southern Medical University Animal Centre (Guangzhou, China), maintained under specific pathogen-free conditions, and fed autoclaved mouse chow and tap water ad libitum. All animal experiments were approved by the local institutional animal committee of the Southern Medical University and were performed in accordance with Chinese animal protection laws.

### 2.2. Conditioning Doses

Bu and Cy were purchased from Sigma-Aldrich (St. Louis, MO, USA), and Flu was obtained from Bayer (Leverkusen, Germany). Bu was dissolved in DMSO at 40 mg/mL and dispersed in phosphate-buffered saline. Cy and Flu were dissolved in sterile water at 10 mg/mL. Based on previous reports [12–14], BALB/c recipient mice received daily intraperitoneal injections of Bu from day −7 to day −4 (25 mg/kg/d or 20 mg/kg/d), followed by injection of Cy from day −3 to day −2 (100 mg/kg/d or 150 mg/kg/d), or Flu from day −6 to day −2 (100 mg/kg/d, 50 mg/kg/d or 20 mg/kg/d) followed by Bu from day −5 to day −2 (25 mg/kg/d). Day −1 was the resting day. All conditioned mice were transplanted with 2 × 10^7^ bone marrow cells combined with 2 × 10^7^ splenocytes from allogeneic (C57BL/6) donors at day 0. We then recorded the survival of recipients after transplantation to determine the optimal dose of the conditioning agents.

### 2.3. Transplantation

Donor mice were killed by cervical dislocation after anesthesia. Bone marrow cells were prepared by flushing femurs and tibias with RPMI-1640. Splenocytes were prepared by passing spleens through cell strainers. After conditioning with the determined optimal doses, recipients were intravenously injected with 2 × 10^7^ bone marrow cells combined with 2 × 10^7^ splenocytes from either allogeneic (C57BL/6) or syngeneic (BALB/c) donors at day 0.  Table 1 summarizes the groups.

### 2.4. aGVHD Monitoring

The general situation and survival of recipient mice were monitored and recorded daily for 45 days after transplantation. Metrics included weight loss, posture, activity, fur texture, and skin integrity. A clinical aGVHD score was performed on the basis of these five symptoms according to a published staging system [15].

### 2.5. aGVHD Pathologic Scoring

Small intestine, skin, spleen, and liver samples of recipients were collected on day +14 after transplantation and were fixed in 10% formalin, embedded in paraffin, sectioned, slide mounted, and stained with a hematoxylin and eosin (HE) staining kit (Thermo Scientific, Boston, MA, USA). The samples were scored by an experienced pathologist blinded to the treatment groups on the basis of the previously reported histopathology scoring system [16].

### 2.6. Chimerism and Cell Subset Analyses

Analyses of chimerism and cell subset composition were performed by flow cytometry. Lymphocytes isolated from bone marrow and spleen were washed and resuspended in FACS buffer. The isolated lymphocytes were incubated with anti-mouse monoclonal antibodies against CD4-Percp-Cy5.5, CD8-PE, CD3-PE, CD19-APC, MHC Class I (H-2Kb)-FITC, and MHC Class I (H-2Kd)-APC (eBioscience, San Diego, CA, USA) for 30 min at 4°C in the dark. The cells were then washed twice with FACS buffer and analyzed on a BD Flow cytometer (BD Biosciences, San Jose, CA, USA). The percentage of donor chimerism was calculated according to the following equation: donor chimerism = [donor cells/(host cells + donor cells)] × 100% [17].

### 2.7. Cytokine Measurements

Mice were killed by exsanguination after anesthesia on days +3, +5, +7, and +14 after transplantation. Blood samples were collected and centrifuged at 400 g for 10 min, and the serum supernatant was harvested for analysis by enzyme-linked immunosorbent assay (ELISA) kits for quantitative detection of murine IL-4 and IFN-*γ*, according to manufacture protocols. Microwell strips were analyzed with a microwell reader (Molecular Devices, Sunnyvale, CA, USA).

### 2.8. Statistical Analyses

All data were analyzed using SPSS 19.0 (SPSS, Chicago, IL, USA). Data are presented as mean ± standard error of the mean (SEM). The Kaplan-Meier and Log rank tests were used to analyze survival data. The aGVHD clinical and pathological scores were compared between groups conditioned with Bu-Cy or Flu-Bu using the nonparametric unpaired Mann–Whitney *U* test. Concentration of cytokines and the proportion of CD3^+^, CD19^+^, CD4^+^, and CD8^+^ cells were tested for normality by applying the Shapiro-Wilk test. If normality was given, an unpaired *t*-test (two-sided) was applied. If the data did not meet the criteria for normality, the nonparametric unpaired Mann–Whitney *U* test was applied. Statistical significance for all analyses was established when *P* value was less than 0.05.

## 3. Results

### 3.1. Optimal Conditioning Doses

**Figure 1:**
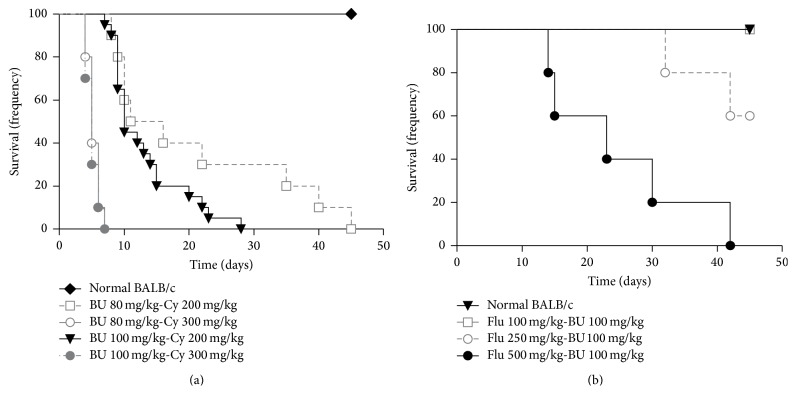
Survival analysis of different conditioning doses. Male BALB/c were conditioned with different doses of Bu-Cy or Flu-Bu and transplanted with 2 × 10^7^ bone marrow cells and 2 × 10^7^ splenocytes from C57BL/6. (a) Survival analysis of mice conditioned with Bu-Cy. (b) Survival analysis of mice conditioned with Flu-Bu.

All recipients conditioned with Bu (80 mg/kg)-Cy (300 mg/kg) or Bu (100 mg/kg)-Cy (300 mg/kg) died within 7 days after transplantation. The median survival of recipients conditioned with Bu (80 mg/kg)-Cy (200 mg/kg) was 13.5 days, with 30% of the mice surviving more than 35 days. Meanwhile, allogeneic transplanted mice after conditioning with Bu (100 mg/kg)-Cy (200 mg/kg) died within 30 days after transplantation (median survival = 10 days) (Figure 1(a)). All recipients conditioned with Flu (100 mg/kg)-Bu (100 mg/kg) and more than 60% of recipients conditioned with Flu (250 mg/kg)-Bu (100 mg/kg) survived over 45 days. In addition, the median survival of mice conditioned with Flu (500 mg/kg)-Bu (100 mg/kg) was 23 days, and all died within 45 days (Figure 1(b)). The survival time of allogeneic recipients in Bu (100 mg/kg)-Cy (200 mg/kg) group or Flu (500 mg/kg)-Bu (100 mg/kg) group was uniform.

### 3.2. Conditioning Regimens Affect the Severity of aGVHD

**Figure 2:**
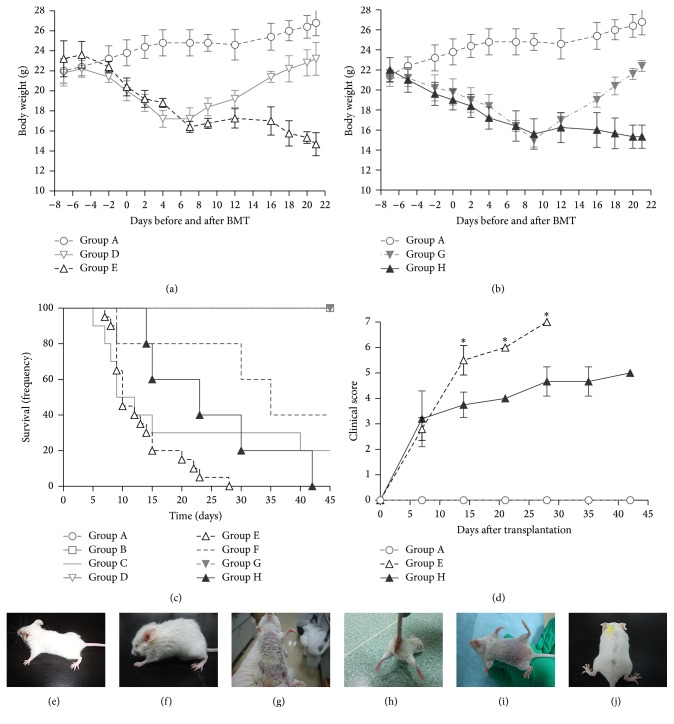
aGVHD manifestations, survival, and aGVHD clinical scores. (a) Weight loss started after conditioning and bone marrow transplantation in allogeneic and syngeneic groups conditioned with Bu-Cy. (b) Weight loss started after conditioning and bone marrow transplantation in allogeneic and syngeneic groups conditioned with Flu-Bu. (c) Survival analysis of different treatment groups. (d) aGVHD clinical scores of allogeneic recipients. (e) Appearance of mice in Group A. (f–h). Appearance of allogeneically transplanted mice (conditioned with Bu-Cy). (i-j) Appearance of allogeneically transplanted mice (conditioned with Flu-Bu). Group A: Normal BALB/c in the same condition; Group B: BALB/c injected intraperitoneally with 0.0625 mL DMSO; Group C: BALB/c conditioned with Bu (100 mg/kg)-Cy (200 mg/kg); Group D: BALB/c conditioned with Bu (100 mg/kg)-Cy (200 mg/kg) and transplanted with 2 × 10^7^ bone marrow cells, 2 × 10^7^ spleen cells from BALB/c; Group E: BALB/c conditioned with Bu (100 mg/kg)-Cy (200 mg/kg) and transplanted with 2 × 10^7^ bone marrow cells, 2 × 10^7^ spleen cells from C57BL/6; Group F: BALB/c conditioned with Flu (500 mg/kg)-Bu (100 mg/kg); Group G: BALB/c conditioned with Flu (500 mg/kg)-Bu (100 mg/kg) and transplanted with 2 × 10^7^ bone marrow cells, 2 × 10^7^ spleen cells from BALB/c; Group H: BALB/c conditioned with Flu (500 mg/kg)-Bu (100 mg/kg) and transplanted with 2 × 10^7^ bone marrow cells, 2 × 10^7^ spleen cells from C57BL/6. All data are presented as mean ± s.e. for 3–7 animals in each group per time point. ^*∗*^ *P* < 0.05.

Allotransplanted mice conditioned with Bu-Cy developed typical manifestations of aGVHD after transplantation, including weight loss, mental fatigue, low mobility, diarrhea, hunched posture, and fur loss (Figures 2(a) and 2(f)–2(h)). These symptoms were not as severe in the group conditioned with Flu-Bu (Figures 2(b), 2(i), and 2(j)). Except for day +7 (*P* = 0.606), the aGVHD clinical scores for allogeneic recipients conditioned with Bu-Cy were higher than Flu-Bu conditioned recipients on days +14 (*P* = 0.017), +21 (*P* = 0.046), and +28 (*P* = 0.034) (Figure 2(d)). Compared with Bu-Cy conditioning (median survival = 10 days), Flu-Bu conditioning (median survival = 23 days) showed significantly longer survival (*P* = 0.012) (Figure 2(c)). Syngeneic recipients showed minor changes in weight loss and low mobility, but they reached a stable body weight within 21 days after transplantation (Figures 2(a) and 2(b)) and survived more than 45 days (Figure 2(c)). There was no significant difference (*P* = 0.27) in survival between nontransplanted BALB/c mice that underwent chemotherapy with Bu-Cy and Flu-Bu (Figure 2(c)).

### 3.3. Pathological Changes of aGVHD

**Figure 3:**
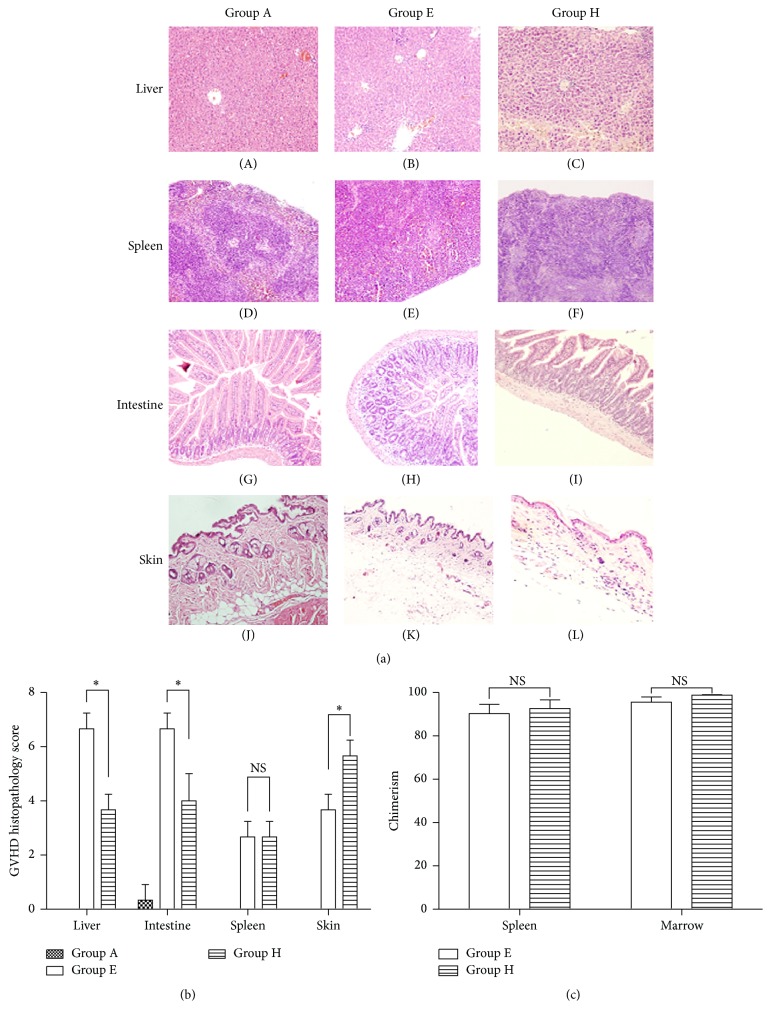
Pathologic changes of aGVHD and chimerism analysis. (a) Pathologic changes of liver, spleen, intestine, and skin on day +14 after transplantation. (b) Pathologic scores for liver, spleen, intestine, and skin on day +14 after transplantation. (c) Bone marrow and spleen chimerism of allogeneic recipients on day +14 after transplantation. Group A: normal BALB/c in the same condition. Group E: BALB/c conditioned with Bu (100 mg/kg)-Cy (200 mg/kg) and transplanted with 2 × 10^7^ bone marrow cells, 2 × 10^7^ spleen cells from C57BL/6. Group H: BALB/c conditioned with Flu (500 mg/kg)-Bu (100 mg/kg) and transplanted with 2 × 10^7^ bone marrow cells, 2 × 10^7^ spleen cells from C57BL/6. All data are presented as mean ± s.e. for 3–7 animals in each group per time point. ^*∗*^ *P* < 0.05; NS, not significant, *P* > 0.05.

Pathologic signs, including destruction of normal structures and inflammatory infiltration, were detected in target organs such as liver, spleen, skin, and intestine of the allogeneic recipients conditioned with Bu-Cy or Flu-Bu by HE staining on day +14 after transplantation (Figure 3(a)). Compared with Flu-Bu, the pathologic scores in the liver and intestine of Bu-Cy were significantly higher, with *P* = 0.043 and *P* = 0.046, respectively, while the score in skin was on the contrary, with *P* = 0.043. In addition, there was no statistically significant difference (*P* = 1.0) between the pathologic score in the spleen of Bu-Cy and Flu-Bu conditioned mice (Figure 3(b)).

### 3.4. Chimerism of Allogeneic Recipients

As shown in Figure 3(c), allogeneic recipients conditioned with Bu-Cy or Flu-Bu all reached full bone marrow (95.55% ± 2.42% and 98.76% ± 0.30%, resp.) and spleen (90.30% ± 4.22% and 92.59% ± 4.05%, resp.) chimerism at day +14 after transplantation. Furthermore, a significant difference was not observed in bone marrow (*P* = 0.12) or spleen (*P* = 0.44) chimerism between Bu-Cy and Flu-Bu conditioning.

### 3.5. Cytokine Concentration after Allo-HSCT

**Figure 4:**
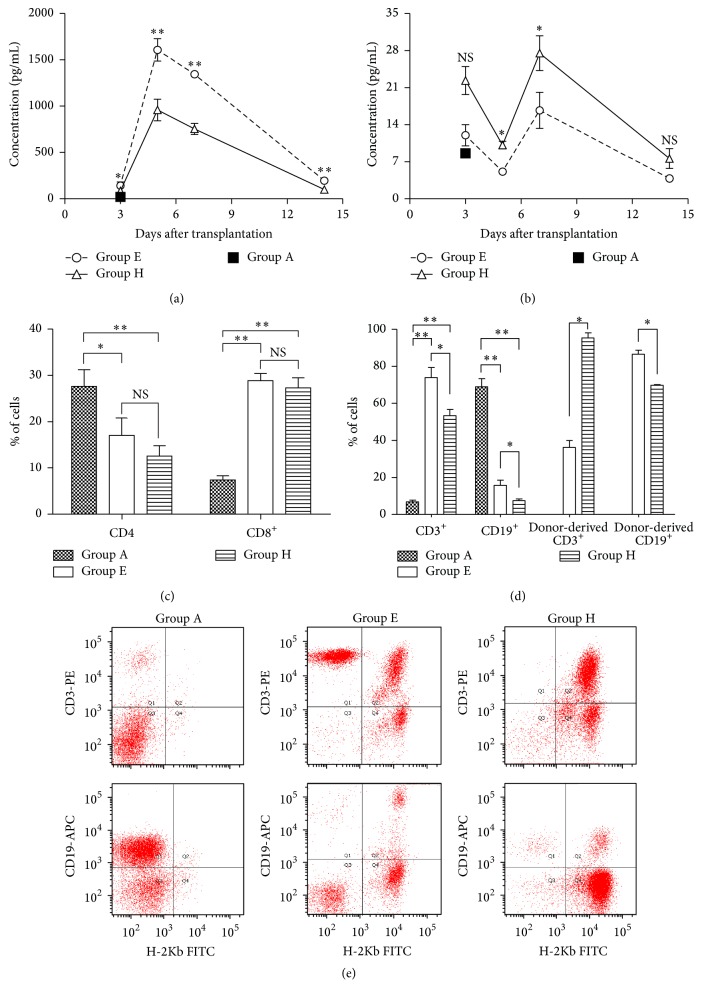
Expression of IFN-*γ* and IL-4 in serum on days +3, +5, +7, and +14 after allo-HSCT, proportion of CD3^+^ and CD19^+^ cells in bone marrow and CD4^+^ and CD8^+^ cells in spleen 14 days after transplantation. (a) INF-*γ* concentration in serum on days +3, +5, +7, and +14 after allo-HSCT. (b) IL-4 concentration in serum on days +3, +5, +7, and +14 after allo-HSCT (c) Proportion of CD4^+^ and CD8^+^ T-cells in spleen 14 days after allo-HSCT. (d, e) Proportion of CD3^+^ and donor-derived CD3^+^ T-cells and CD19^+^ and donor-derived CD19^+^ B-cells in bone marrow 14 days after allo-HSCT. Group A: normal BALB/c in the same condition. Group E: BALB/c conditioned with Bu (100 mg/kg)-Cy (200 mg/kg) and transplanted with 2 × 10^7^ bone marrow cells, 2 × 10^7^ spleen cells from C57BL/6. Group H: BALB/c conditioned with Flu (500 mg/kg)-Bu (100 mg/kg) and transplanted with 2 × 10^7^ bone marrow cells, 2 × 10^7^ spleen cells from C57BL/6. All data are presented as mean ± s.e. for 3–7 animals in each group per time point. ^*∗*^ *P* < 0.05; ^*∗∗*^ *P* < 0.01; NS, not significant, *P* > 0.05.

We next analyzed the expression of IFN-*γ* and IL-4 in the serum of allotransplanted mice on days +3, +5, +7, and +14 after transplantation comparing Bu-Cy with Flu-Bu conditioning. INF-*γ* concentrations all reached a maximum on day +5 in the Bu-Cy (1607.78 ± 119.53 pg/mL) and Flu-Bu (959.04 ± 116.09 pg/mL) conditioning, and these values were significantly different (*P* = 0.001). The differences in serum IFN-*γ* levels remained significant on days +7 (*P* = 0.001) and +14 (*P* = 0.002) (Figure 4(a)). In contrast, on day +5 after transplantation, serum IL-4 levels in recipients conditioned with Bu-Cy (5.13 ± 0.09 pg/mL) and Flu-Bu (10.14 ± 0.73 pg/mL) reached the lower limit, and these values were significantly different (*P* = 0.007). The IL-4 concentration in Bu-Cy conditioned recipients remained lower (*P* = 0.038) than Flu-Bu conditioned recipients on day +7 (Figure 4(b)).

### 3.6. Bone Marrow Involvement in aGVHD after Allo-HSCT

Compared with normal BALB/c mice, a strong increase in the proportion of CD3^+^ T-cells and a significant decrease in the CD19^+^ B-cells were observed in the bone marrow of all allogeneic recipients at day +14 after transplantation. Meanwhile, the frequency of CD3^+^ T-cells of recipients receiving Bu-Cy conditioning (73.97% ± 5.37%) was significantly higher compared to mice receiving Flu-Bu conditioning (53.37% ± 3.31%, *P* = 0.005). In addition, >90% of the infiltrated T-cells in the Flu-Bu group were of donor origin, which was significantly higher than Bu-Cy group (36.17% ± 3.82%, *P* = 0.000) (Figures 4(d) and 4(e)). In contrast to CD3^+^ T-cells, there was also a significant difference (*P* = 0.008) in the bone marrow CD19^+^ B-cell proportion between allogeneic recipients conditioned with Bu-Cy (15.73% ± 2.75%) and Flu-Bu (7.47% ± 1.00%). The donor-derived CD19^*+*^ B-cell proportion in Bu-Cy was higher than Flu-Bu (*P* = 0.000) (Figures 4(d) and 4(e)). Furthermore, compared to normal BALB/c mice, the subset of splenic lymphocytes in the allogeneic mice revealed a strong decrease in CD4^+^ T-cells (*P* = 0.025 and *P* = 0.004 in Bu-Cy and Flu-Bu, resp.) and a strong increase in CD8^+^ T-cells (*P* = 0.000 and *P* = 0.000 in Bu-Cy and Flu-Bu, resp.), which resulted in a decrease in the CD4^+^/CD8^+^ T-cell ratio. However, no significant differences were detected in the proportion of CD4^+^ and CD8^+^ T-cells between Bu-Cy and Flu-Bu (Figure 4(c)).

## 4. Discussion 

aGVHD is a fatal complication of allo-HSCT [18]. To mimic the clinical situation, we developed a mouse model of aGVHD conditioned with Bu-Cy or Flu-Bu.

Previous publications have shown that Bu treatment with 100–150 mg/kg is enough to induce myeloablation and full donor chimerism [12, 19]. In addition, another study has demonstrated that the metabolism and pharmacokinetics of Fludarabine differed between humans and animals and that the maximum tolerated doses of Fludarabine were 10 to 30 times lower in humans than in mice [13]. Based on these findings, we chose the different doses of Bu-Cy and Flu-Bu for the experimental treatment. We found that increasing the dose of Cy (300 mg/kg) or decreasing the doses of Bu (80 mg/kg) and Flu (100 or 250 mg/kg) resulted in quick death or inhomogeneous survival of the mice. Recommended from previous publications [12, 20], we determined Bu (100 mg/kg)-Cy (200 mg/kg) and Flu (500 mg/kg)-Bu (100 mg/kg) to be the optimal conditioning doses. After transplantation, the syngeneic recipients did not develop clinical manifestations of aGVHD and survived more than 45 days. Moreover, there was no significant difference in the survival of mice conditioned with Bu-Cy or Flu-Bu without transplantation. Thus, the direct toxicity of the two conditioning regimens in our study was limited, and the difference between them was indistinguishable.

Li et al. [21] have shown that helminth infection can alleviate aGVHD with preserved antitumor immunity by regulating immunity directly or through modulation of gut flora, which demonstrated that infestations have an important role in the immunological modulation of aGVHD. Moreover, aGVHD-related mortality was significantly reduced in germ-free mice and in conventional animals after antibiotic treatment [22]. To exclude the impacts of infestations, all mice in our study were maintained under specific pathogen-free conditions and the whole experiment was completed in sterile environment.

The conditioning regimen is a pivotal factor for the development of aGVHD [23]. In Bouazzaoui et al.'s study, Flu-Bu conditioning resulted in a delayed aGVHD and improved survival compared to TBI conditioning [20]. In our study, all allotransplanted mice conditioned with Bu-Cy or Flu-Bu developed lethal aGVHD with typical manifestations and histopathological changes starting from day +7. We also found that the conditioning regimen of Flu-Bu resulted in less severe symptoms and a longer survival time compared with Bu-Cy. These results are in line with phase 3 trial reported by Rambaldi et al. [11], in which nonrelapse mortality and incidence of grades III-IV acute graft-versus-host disease were significantly reduced in the Flu-Bu group compared with the Bu-Cy group. Compared with the publication of Sadeghi et al. [14], in which all mice died within 60 days (median survival = 11 days) after transplantation, the Bu-Cy conditioning regimen in our study resulted in more uniform survival of aGVHD mice. Of note, Riesner et al. [24] have showed a delayed aGVHD in a murine MHC-matched, miHA-mismatched model [LP/J (H2k^b^) →C57BL/6(H2k^b^)] using Bu (100 mg/kg)-Cy (300 mg/kg) conditioning. These discrepancies between our study and previous publications may be due to the degree of allomismatch (MHC-mismatched or miHA-mismatched), differences in graft (bone marrow cells and splenocytes), and the intensity of conditioning.

Pretransplant conditioning damages host tissues, causing recipient tissues to secrete proinflammatory cytokines and amplify antigen-presenting cells in response to the tissue damage [25]. After interacting with antigen-presenting cells, CD4^+^ T-cells can activate and differentiate into Type 1 T helper cells (Th1) and Type 2 T helper cells (Th2), depending on the cytokine milieu. Cytokines play an important role in the development of aGVHD [26]. As a classical Th1-derived cytokine, IFN-*γ* is crucial for the proliferation of cytotoxic T-cells [27], while IL-4 plays an important role in the differentiation of CD4^+^ Th2 cells [28]. Th1/Th2 polarization of T helper cell subsets affects the development of GVHD, determining end organ damage [29]. Previous studies have shown that, in a mouse model of aGVHD, damage to the lung and liver was mainly mediated by Th1 cells, while damage to the skin was mainly mediated by Th2 cells [30]. In our study, hepatic and intestinal tissue damage in the aGVHD mice conditioned with Bu-Cy was more severe compared with Flu-Bu conditioned mice, similar to the overall manifestations of aGVHD. These results may be supported by the discovery of a significant increase of IFN-*γ* and decrease of IL-4 expression in the serum of recipients conditioned with Bu-Cy compared to those conditioned with Flu-Bu. Therefore, we suggest that the Flu-Bu conditioning regimen resulted in less severe aGVHD and pathologic changes in the liver and intestine through decreased IFN-*γ* secretion and increased IL-4 secretion compared with the Bu-Cy conditioning regimen. This is also consistent with the possibility that increased IL-4 expression may be a mechanism to reduce aGVHD depended upon by some immunocytes such as NKT cells and basophils [28, 31].

As is known, Natural Killer (NK) cells are important participants in immune reactions. Previous murine studies have demonstrated that donor NK cells can suppress the development of aGVHD while inducing an antitumor response [32]. The primary effector function of NK cells is to eliminate susceptible target cells and amplify the antitumor immune response by direct cellular lysis and cytokine production [33]. Whether NK cells play a part in the observed differences of the frequency and severity of aGVHD in our study will be one of our next investigation contents.

Recently, bone marrow has been identified as a target organ of aGVHD [34, 35]. Shono et al. [36] have demonstrated the destruction of bone marrow hematopoietic niches by donor T-cells in murine models of aGVHD, resulting in bone marrow suppression, including B lymphopoiesis, and this phenomenon was identified as bone marrow GVHD. Cell subset analysis showed greater CD3^+^ T-cells infiltration in bone marrow of allogeneic recipients conditioned with Bu-Cy compared to Flu-Bu, while the donor-derived CD3^+^ T -cell data showed the opposite. In addition, the proportion of the total CD19^+^ cells and of donor-derived CD19^+^ B-cells were all significantly higher in the bone marrow of recipients conditioned with Bu-Cy, compared to those conditioned with Flu-Bu. This leads to the conclusion that the Flu-Bu conditioning regimen resulted in higher donor-derived CD3^+^ T-cells infiltration and more severe impairment of B-cell reconstitution in bone marrow. The conclusion was also supported by Haddad et al. [37, 38], who showed that the type of conditioning regimen was related to the impairment of B-cell lymphopoiesis. Moreover, all allogeneic recipients revealed the impact of aGVHD on immune reconstitution, resulting in a decreased splenic CD4^+^/CD8^+^ T-cell ratio, and there was no significant difference between recipients conditioned with Bu-Cy and Flu-Bu. This suggests that bone marrow involvement is independent of the severity of aGVHD, which is in line with clinical observations that there is not necessarily a relationship between impaired B-cell reconstitution and worse outcomes [39]. Furthermore, it has been considered that increased donor-derived T-cell infiltration in bone marrow may be beneficial for GVL, which is important for the prevention of relapse [40]. The subsequent effects of impaired B-cell reconstitution and donor-derived T-cell infiltration require further investigation.

## 5. Conclusions

This study reports a murine model of aGVHD conditioned with Bu-Cy or Flu-Bu. Our results demonstrate that conditioning regimens play a crucial role during the development of aGVHD. Cyclophosphamide worsens aGVHD, while Fludarabine aggravates bone marrow damage. This indicates that bone marrow involvement has no necessary relationship with the severity of aGVHD. Our findings will help us understand the different impacts on aGVHD of different conditioning regimens and provide a platform for preclinical investigations of aGVHD.

## Figures and Tables

**Table 1:** Experiment mouse groups.

Experimental groups	Conditioning regimens	Graft	Number of mice
Group A	—	—	5
Group B	—	—	5
Group C	Bu-Cy	—	10
Group D	Bu-Cy	2 × 10^7^ BMC + 2 × 10^7^ SPC from BALB/c	10
Group E	Bu-Cy	2 × 10^7^ BMC + 2 × 10^7^ SPC from C57BL/6	20
Group F	Flu-Bu	—	10
Group G	Flu-Bu	2 × 10^7^ BMC + 2 × 10^7^ SPC from BALB/c	10
Group H	Flu-Bu	2 × 10^7^ BMC + 2 × 10^7^ SPC from C57BL/6	10

Group A: normal BALB/c mice in the same condition; Group B: BALB/c mice injected intraperitoneally with 0.0625 mL DMSO.
